# How do students configure the school psychologist? Ethnopsychology of the Italian school post-COVID and implications on well-being and health

**DOI:** 10.3389/fpsyg.2025.1655423

**Published:** 2025-12-10

**Authors:** Antonio Iudici, Federica Epifani, Marianna Belli

**Affiliations:** 1 Department of Philosophy, Education, Sociology and Applied Psychology of Padua (FISPPA), University of Padua, Padua, Italy; 2 Institute of Psychology and Psychotherapy, Padua, Italy

**Keywords:** school psychologist, psychological service, school well-being, health, ethnopsychology

## Abstract

**Introduction:**

The research project aims to explore the discourses and beliefs that students have towards the school psychologist. Recent historical and cultural events have brought to light existing difficulties and needs, due to the lack and inadequacy of resources, revealing a clear separation between the school context and that of daily life. The urgency and necessity of a coordinated psychological intervention that moves between the various levels of the school have been highlighted.

**Method:**

The research involved 70 high school students from various institutions. Through Harré’s discursive positioning analysis, it was possible to detect the configuration that students have regarding themselves and the school psychologist, and, above all, to understand the dynamics of the school community, which are reflected in the interaction between actors, creating particular scenarios of reality.

**Results:**

As a common background to the narratives, the image of the school psychologist emerged as a fundamental and necessary professional figure, whose constant presence in the school context is required. At the same time, various representations of the functions that the psychologist can assume emerged. The psychologist’s intervention proves useful not only in emergency situations and difficulties where moments of impasse and suffering manifest, but especially in the moments and events of daily life that take place at school through the promotion of well-being and support for adolescents.

**Discussion:**

This study is particularly important in the current context because by giving voice to students’ experiences and needs, it is possible for the school system to address and manage them. The research directs its usefulness to professionals who intend to carry out their work in the school context, offering important information on how to build an adequate psychological service at school.

## Introduction

The unique historical and cultural context of recent years, characterized by events such as the pandemic and the current conflicts in Ukraine and the Middle East, has highlighted the impact that current events can have on educational contexts (Reimers and Schleicher, 2020). Consequently, attention has been focused on how students interpret the uncertainties arising from an unstable situation (Commodari and La Rosa, 2021). Some studies have shown that it is not always easy for students to communicate their distress or openly discuss certain topics (Smorti, 2007; Guessoum et al., 2020), just as in other cases, current school personnel are not always inclined to address themes such as violence, war, and armed conflicts (Brock and Cowan, 2004; Vicari et al., 2022). It is also known that the pandemic has left lingering effects on some students, who were forcibly compelled to interrupt various social, educational, and sports activities (Loades et al., 2020; Lingiardi, 2021). When schools fail to address students’ emotions or distress, a rift can develop between the school context and that of daily life (Poulou, 2017; Moro, 2021; Zins et al., 2004). One of the tools available to schools to intercept student distress is certainly the role of the psychologist, who operates in schools in a recognized manner in most European contexts (Rens et al., 2018; Schleicher, 2018).

Analysis of studies in the literature has revealed that psychology in the school context has adapted and responded to the needs of the times: it has addressed the demand for social equality, issues related to psychotropic substance use and deviance, the phenomenon of school dropout, health promotion, and teacher training (Dentici, 1998).

In recent times, schools have primarily attributed to psychologists a merely emergency and sometimes curative function, with actions that involve treating established problems (Cornoldi and Molinari, 2019). Historically, interventions and methods typical of clinical psychology have prevailed, whose application was required for moments when urgency manifested.

The image of the psychologist that has been configured over time has been that of one “who should solve the problems of ‘discomfort,’ with interventions on individual cases or with group meetings with students or teachers” (Trombetta et al., 2008).

The focus of interventions and research has thus been directed towards particular and symptomatic situations of students in difficulty, and through their identification and evaluation, has concentrated on risk factors inherent to both health and dysfunctional behaviors such as bullying (Dentici, 1998; Menesini and Salmivalli, 2017). All this has led to neglecting all aspects concerning the “school” organization in the complex network of interactions in which it is embedded (Lucisano et al., 2018).

Subsequently, attention has been placed more on interactive processes involving the relationship between all roles involved (Capurso and Dennis, 2017; Mattioli, 2022), focusing on observing those dynamic processes that take place in the school context and are characterized by an interweaving of relationships, discourses, and meanings. This evolution has led to a more systemic view of the school psychologist’s role, as highlighted by Sheridan and Gutkin (2000), who emphasize the importance of considering the entire school ecosystem in psychological intervention practices. The school, therefore, has for several years been viewed not as a possible container for discomfort prevention interventions, but rather as an agent and promoter of well-being (Iudici, 2014).

Thus, alongside realities that operate on urgency, projects and interventions are being added that range from risk prevention to health promotion: there is a shift from a perspective of care, rehabilitation, and repair to a perspective of protection and safeguarding, to be implemented through information, increasing positive factors, and anticipating scenarios that may configure in a given context (Meyers and Meyers, 2003). Moreover, the emergency logic can be replaced only when stable connections are created in the territory, including social and health services, in which the school positions itself as an essential reality open to all citizens (Anderson-Butcher and Ashton, 2004).

As is known, Services are effective to the extent that they respond to precise user needs. A disconnect between Service and user needs creates dissatisfaction with the service or low use of the service, with consequent waste of resources. Also for this reason, we asked ourselves whether, in this period of uncertainty, the need to interact with a psychologist at school is changing. What image do students have of the psychologist at school? What might the needs be today? What requirements could be highlighted?

Specifically, the research objectives focused on how students configure the school psychology service, the role of the school psychologist, and their own role as users; on the needs, expectations, requests, and limitations that students place on the figure of the school psychologist and the modalities through which interaction with the latter can occur.

## The status of service in Italy: ethnopsychological elements

Contemporary Italian schools are facing a series of challenges and changes that reflect the social and cultural transformations of the country. One of the most evident aspects is the increasing multiculturalism of classrooms, a phenomenon that has taken root in recent decades. This demographic shift has necessitated a reevaluation of certain didactic practices and relational dynamics within classrooms (Santerini, 2010). Traditionally, the relationship between teachers and students has been based on a certain formality, although a more collaborative approach has been emerging recently. This period of change is not without challenges, as it requires a rethinking of the teaching role and didactic methodologies (Cavalli and Argentin, 2010).

A distinctive element of the Italian school system remains the emphasis placed on theoretical learning. While this aspect ensures a solid knowledge base, it has been criticized for potentially neglecting practical skills and soft skills (OECD, 2020a). The evaluation system, still predominantly based on numerical scales, is at the center of a debate on the opportunity to adopt more holistic approaches (Domenici, 2007).

A certainly innovative aspect of Italian schools is their approach to inclusivity, especially regarding students with disabilities. Italy has been a pioneer in this field, as documented by D'Alonzo (2008).

The role of the family in the Italian educational system is particularly significant and represents a distinctive characteristic of the country’s school culture. In many institutions, there exists a concept of “educational alliance” between school and family that is rooted in Italian culture. Indeed, parents tend to be very present in their children’s school life, and for some, this can increase student well-being and even school performance (Mantovani, 2012; Pati, 2019). At the same time, Italian families often have high expectations regarding their children’s academic success, which can also be a source of stress for students (Bonica and Sappa, 2010). These expectations also translate into learning support activities, as parents often invest time and resources in participation in extracurricular activities and projects (Gasperoni, 2013). Moreover, the weight of parents in influencing educational choices is decisive both in the choice of direction and with respect to the entire educational path (Colombo, 2017). In many cases, Italian parents play a significant role in mediating with families of immigrant origin, often taking on linguistic and managerial assistance (Santagati, 2015). Concurrently, this intense family involvement can lead to the creation of status differences between families from different social and cultural classes and also complicate the task of teachers who find themselves managing and balancing family demands with those of the school and institutions (Argentin, 2018).

Finally, the impact of academic stress on Italian students cannot be overlooked. Several studies have highlighted how levels of anxiety and stress related to school performance are particularly high among Italian students, raising important questions about psychological well-being in the educational context (Alivernini et al., 2019).

Regarding school psychology, Italy is currently the only European country that has not yet officially recognized the professional figure of the school psychologist, whose work is limited by the demands of the moment and the availability of resources. The lack of specific legislation, in fact, does not allow for clear references regarding the areas, methods of intervention, and evaluation of the effectiveness of the intervention. This historical moment thus offers us an opportunity to comprehensively delineate the complexity of the professional figure of the school psychologist and their work (Cornoldi and Molinari, 2021).

A survey (Istituto Piepoli, 2021) highlighted that 81% of the Italian population requests a psychologist at school, reaching 94% in the age range between 15 and 18 years. Among the fundamental activities, those most frequently indicated in the interviews were listening and support at 54%, prevention of discomfort at 41%, support for families at 29%, and consultation to the school system as a whole and support for teachers at 18%. Seven out of ten students between 15 and 18 years old preferred the term ‘listening and support’. This data denotes greater importance to students’ experiences regarding the school experience and the close links between these experiences and adaptation and success in the academic field. At present, there are some regional experiences, but there is no national law regulating activities, although at the experiential level, the presence of psychologists is very widespread in the role of occasional consultant.

## Method

### Theoretical background

This research draws upon the premises of the interactionist paradigm, within which individuals ascribe meaning to their experiences and organize their conduct through symbolic and linguistic processes (Berger and Luckmann 1969; Iudici and Fabbri, 2017; Romania, 2012).

In symbolic interaction, individuals indeed give life to identities and roles, and negotiate rules and meanings (Salvini and Dondoni, 2011). It is possible to grasp the meanings of language through the modalities in which it is used in everyday life contexts: observing how a phrase is employed allows for the detection of its sense, as it is language that shapes what is considered “real” (Wittgenstein, 2017). Consequently, the meanings of words are not essential but rather positional, and they change based on the place they occupy in each actor’s current perspective. People’s narratives are expressions of how things stand for the subject (Harré and Gillett, 1994). In line with what has been expressed thus far, this research has focused attention on students’ narratives. Through the analysis of these narratives, it has been possible to access the meanings and reality configurations of the students themselves in reference to the research objectives. This research can assist various schools that are currently considering implementing a School Psychology Service through the study of student narratives, in order to respond precisely to the needs and requirements of the students.

### Participants

The research involved 70 secondary school students, aged between 14 and 19 years, comprising 36 females, 29 males, and 5 who chose not to identify their gender. The students are from various institutions, including 14 from the Scientific and Classical Lyceum of Ostuni; the Higher Institute of Higher Education of Castel Volturno; ISISS of Sessa Aurunca; Lyceum of Rimini; Institute of Higher Education of Camposampiero, and the Institute of Higher Education of Melzo, respectively located in the Puglia, Campania, Emilia-Romagna, Veneto, and Lombardy regions.

This study was approved by the Ethics Committee of the University of Padua (n. 1,367).

### Aims

The main research objectives have been outlined as follows:

1. Configuration of the school psychologist’s role: explore discursive and narrative processes to identify configurations regarding the role of the psychologist in school;
2. Configuration of the user’s role and requests: explore discursive and narrative processes to investigate the configuration of the user’s request in relation to the school psychologist;
3. Configuration of students’ current needs: explore discursive and narrative processes to identify the ways in which students configure their current needs and requirements, both in relation to the work of the school psychologist and to the historical-cultural period in which they are immersed.

### Investigation methodology and data collection

The open-ended questionnaire is a qualitative research tool that allows participants to respond freely, using their own words, without being constrained by predefined options (Braun et al., 2020). When used online, this method offers several advantages, including the ability to reach a large number of geographically dispersed participants and the convenience for respondents to complete the questionnaire at times and places most suitable for them (Lefever et al., 2007).

The construction of the questionnaire requires particular attention to the selection and definition of questions, which must be clear, unambiguous, and capable of stimulating rich and detailed responses (Krosnick, 2018). It is also important to consider the number of questions, which should not be excessive to avoid participant fatigue (Dillman et al., 2014). In our case, the administration was carried out through the specialized platform Google Forms, as it offers various functionalities for data collection (Regmi et al., 2016) and ensures the privacy and security of participants’ data, in compliance with current regulations such as GDPR in Europe (Buchanan and Hvizdak, 2009).

One of the main advantages of this method is the richness and depth of information it can provide, allowing for the exploration of participants’ perceptions, experiences, and opinions in detail (O'Cathain and Thomas, 2004).

The research commenced with a preliminary investigation conducted through discussions with young students regarding topics of interest and the needs they brought forth and encountered. Following the emergent narratives, a questionnaire was developed as an investigative tool to pursue the objectives outlined thus far, through the collection of narratives derived from responses to questions posed to the students in written form.

This instrument was preferred as it allowed participants a greater degree of freedom regarding the possibility of choosing the moment and time to dedicate to the activity. Another advantage noted was attributing to students the responsibility in choosing to participate in the research, offering their contribution on themes that primarily involve them. This format allowed for the maintenance of anonymity.

Data collection occurred in the period from October 2022 to April 2024 through the snowball effect technique. It was necessary to establish contact with school principals who, upon learning the motivations that promoted the exploratory investigation, showed interest in the project and were inclined to involve teachers. Through word-of-mouth communication with colleagues and students, they proposed to interested students to participate in the research.

The involved principals, designated roles, and teachers were provided with a link through which to access the form containing the questionnaire prepared for collecting and recording responses, composed of eight open-ended questions.

The choice to favor open-ended questions allowed students to provide non-predetermined answers but rather to argue through their own words, fostering the possibility of identifying the respondent’s thoughts and experiences in depth.

### Data analysis and coding

For data analysis, Harré’s (1990) theory of discursive positioning was employed, which allowed for the investigation of the ways in which individuals construct their own story, and thus their sense of self and others. The Positioning framework, developed by Davies and Harré (1990), Harré and van Langenhove (2010), and Harré and Moghaddam 2003, is one of the most widely used tools for discourse analysis and focuses on how individuals and social actors position themselves within discursive contexts. Positioning theory highlights the performative aspect of language, in which speakers use discourse not only to describe but also to enact and reinforce social positions and relationships. This methodology concentrates on the linguistic structures present in texts and consequently goes beyond mere content analysis. Positioning Analysis explores how language is used to construct specific versions of reality and to influence perceptions, beliefs, and actions (Harré and Gillett, 1994). Central to this method is the recognition that language plays an active role in the construction and maintenance of social reality and specific discursive configurations. The individual, thus understood as a discursive agent, through participation in discursive practices and through linguistic utterances, “positions” themselves within a specific location system composed of four coordinates: spatial, temporal, moral, and social (Davies and Harré, 1990).

Indeed, positioning refers to the ways in which roles are assigned, appropriated, or denied to oneself and others, based on the interpretation to which the individual refers. The conceptual tool of Positioning (Harré and van Langenhove, 2010; Davies and Harré, 1990) was used to highlight the dynamic aspects through which the discursive formation of identity occurs, including that of the Italian student in the school context. Positioning Theory asserts that individuals construct their identities and make sense of their social world through the positions they adopt or are assigned within conversations and interactions. These positions are not fixed but are dynamically negotiated through language and discourse. They reflect and shape individuals’ relationships, roles, and identities within specific social contexts (Harré and Moghaddam, 2003; Harré and van Langenhove, 2010).

Positioning analysis paid particular attention to the socio-cultural and ethnopsychological elements of the narratives, recognizing their importance in the discursive construction of the observed reality. This approach highlighted how text and discursive practices not only reflect but actively construct social realities and identities, consistent with the research objective (Iudici et al., 2019; Iudici et al., 2018; Turchi et al., 2022). Discursive positioning tacitly limits the extent of what is logically possible to say and do and adequately delimits a part of the repertoire of possible actions at a given moment in a specific context (Harré and Moghaddam, 2003;). Identifying in students’ narratives the positions they assume and attribute to others can help not only in understanding and identifying the configuration with respect to themselves and the school psychologist but, above all, in understanding the dynamics of the school community, which are reflected in the interaction between actors, creating certain scenarios of reality (Iudici and Renzi, 2015).

Through analysis and positioning, we were able to explore how participants articulate their narratives and positions regarding the area of inquiry: starting, for example, from the configuration of the psychologist, it is possible to anticipate the use of the same role and, in general, to understand who will ask for help and who will not, and for what reasons. Through the configuration of their own needs as students, it is possible to understand which services can actually be organized by institutions to respond to students and create truly useful services.

The derived positionings, of a moral, cultural, and/or institutional order, represent what the cultural matrix, normative references, and institutional role make possible in terms of reality configuration (Harré and van Langenhove, 2010).

The analysis of discursive processes allows for the identification of relevant discourses and positions, thus clarifying their practical implications in participants’ life stories and intervention pathways.

The analytical process involved several distinctive phases:

- A comprehensive examination of texts produced by participants, focused on the research area and its discursive nuances;
- Evaluation within the research team to review the preliminary text analysis, identifying and resolving any discrepancies;
- Definition of fundamental discursive positions in line with the research area and objectives;
- Grouping of findings into key positionings to enhance clarity and facilitate interpretation.

### Validation of scientific data

To enhance the credibility of the research, we explicitly articulated the researchers’ epistemological orientation, delineating the theoretical and conceptual foundations previously described. Familiarity with the data was ensured through preventive collaboration with school principals, who were informed about the study’s aims and objectives.

Data analysis was conducted by all involved researchers, initially individually and subsequently collectively. This approach facilitated a shared and in-depth understanding of the textual data.

The reliability of the results was ensured through detailed explication of data categorization criteria, utilizing a grid of questions and coding parameters related to positioning. This methodology allows for replicability of data collection and analysis procedures. We also provided a precise description of specific data on participants.

The transferability of the study was enhanced by the density of participant responses. Our analytical method, based on the detection of discursive positioning, ensured an accurate reading of the text, thereby increasing the precision of the results. The research’s reliability was further corroborated during the data acquisition and collection phases, as well as in the analysis of the relationship between data and results.

Throughout the research process, we defined specific inquiry protocols for the administered questions. Researchers continuously monitored the application of the method, noting any critical elements as research limitations. During the analysis, responses inconsistent with the objectives of the questions were excluded from the study.

## Results

The significant and representative positionings that emerged from the data analysis are situated along a continuum of interconnected and interdependent placements.

### Configuration of the school psychologist’s role

#### The psychologist as a necessity

One of the most prominent positionings in the first dimension, for the configuration of the school psychologist’s role, was the definitional positioning, through which students established a reality.

For instance, in cases where the psychologist is stated to be “A necessity,” “fundamental,” and “indispensable,” students position the school psychologist as an essential and basic figure for the school system, whose presence is not contestable, assuming their own point of view with obviousness and firmness, as if it included the broader community perspective. The terms employed explicate a linguistic action of certainty and awareness, thus revealing a need and a requirement, and claiming the presence and intervention of the professional figure.

#### The psychologist as mental help/support

Another positioning detected is that of support, through which students have positioned the school psychologist as a professional figure offering their help and support. Through this modality, the school psychologist is associated with the image of a professional capable of alleviating suffering and unraveling the complexity of situations in which students are immersed. For example, in the reported response: “A source of reorientation and a figure of support and clarity that many students need for their mental harmony” (Interv.8), the psychologist is associated with an existential orientation tool.

In the following comment: “A moral support in moments of collapse, a strength to cling to when you are about to give up everything” (Interv.10). The student directs a request for moral support and sustenance to the school psychologist, who is positioned, through the effect of the verb “to cling,” as a “salvific” figure of great strength that can alleviate suffering in moments of great difficulty.

#### The psychologist as a problem solver

As the last significant positioning derived, we present the one that positions the school psychologist as a problem solver. This perspective is firmly assumed in the response “To solve problems” (Int. 26). It is interesting to highlight the linguistic action implemented through the verb “to solve”: it derives the idea that the role of the school psychologist should implement actions that have practical implications in resolving discomfort.

The same verb is used in reference to the noun “dynamic.” It is noteworthy the neutrality of the term compared to those used previously: such as problems, or difficulties.

“I would invite a friend of mine to turn to the school psychologist because I know they might need a psychologist to resolve dynamics that would probably remain in the shadows without the presence of this figure.”

The idea that emerges, in this case, is that the psychologist, with their skills, leads to a greater awareness of lived experiences, bringing a benefit to those who experience them.

### Configuration of requests and the requester

#### The request for support, coaching

In the second dimension, regarding the configuration of requests and the requester, the positioning of consultation was identified, which places the user as in need of help and listening for issues related to daily life stemming from the family and school context, and more generally, for relational dynamics.

For example, in the reported case (where it is stated that the psychologist’s role is “To help them overcome the uncertainties of this age”), the student highlights the need to be supported in a critical moment, that of adolescence, characterized by continuous changes, by a competent person who can facilitate the life journey.

The configuration of the user and their needs moves along a continuum that ranges from the request for help, as just shown, to the request for consultation.

Through the latter, the user assumes an active role, recounting experiences or directly asking for suggestions from the school psychologist.

This last aspect is easily traceable in the students’ responses, reported below, to the question about what a person of the same age might ask a school psychologist: “they could talk about things regarding their family, personal, school experiences, with peers and ask for advice based on what brings them there.”

#### The request not to be judged

The responses belonging to this category describe a scenario where the configuration of the user and the request is influenced and constructed around the possibility of potential prejudices against those who decide to turn to the psychological service or towards the professional offering the service. For example, one student said, “The very first problem is that if you go to the psychologist, then you have to hide the fact that you go there, because you are afraid of being teased more than usual by your classmates; another problem would be that the boy/girl would not even want to try because they might associate the psychologist with a grown-up like a parent; so, they are afraid of what they might say or do and especially of not being understood even by the psychologist” (Int.34).

Another student stated that one of the fears blocking her from turning to the school psychologist is “the shame and judgment about going to the psychologist” (Int.45).

The user, sometimes out of fear and shame of being judged or based on their personal beliefs, presents themselves as one who acts by setting aside their own needs and well-being. The request not to be judged also emerged, as will be highlighted later, from narratives about students’ current needs.

#### The user as normal

Through the definitional positioning, instead, students define the service user through the category of “normality.”

As can be seen from the excerpt “We all need a psychologist in our lives,” the action of turning to a professional who deals with psychological well-being is not understood in the perspective of an exceptional and unusual event, but rather as a socially accepted and widespread reality. The user is thus a subject who, like everyone else, has needs inherent to their psychological life. With this modality, full legitimacy is attributed to the requests.

In conclusion, the case of the response “A normal person if not so how should I describe it” proved interesting, as the student, after referring to the user as a “normal person,” adds a rhetorical question through which they emphasize the evidence and obviousness of such an answer which, therefore, appears as the only conceivable and acceptable one, shared by all.

#### The courageous user

Finally, with the qualifying positioning, a reality is configured in which the user is characterized by a series of peculiarities that distinguish them from those who, instead, decide not to have any confrontation with the school psychologist. In this perspective, those who choose to turn to the professional embody various qualities that make them worthy of admiration and appreciation, expressed, therefore, through positive judgments. The user is associated with the image of a “strong, mature, courageous, determined, intelligent person” and “As a person ready to open up and confront themselves, recognizing their own difficulties and overcoming them, therefore as an open and enterprising person.” With this modality, a change of perspective is felt that dismantles the “fragility” of the person understood in negative and prejudicial terms, to the point of becoming, by becoming aware of it, a characterizing and essential element of the individual. Deciding to face and manage one’s difficulties thus becomes a reason for richness and not weakness.

### Configuration of students’ current needs

#### The need to be legitimized, to be seen not just as numbers and students

Students have configured their current needs through two main positionings. Firstly, they employed a positioning that allowed them to describe their needs through their own point of view, looking at past, present, and future.

As evidenced by the excerpt “After this historical period, I felt the need to go out, see my loved ones (relatives, friends) again, and I needed to return to my old habits,” students’ needs move from considerations aimed at rediscovering and re-establishing lost habits due to historical-cultural changes. The resulting image is that of a return to the past, where it was possible to carry out actions taken for granted, such as going out, taking a walk, seeing friends and relatives, living in community, pursuing one’s passions, in full freedom and carefreeness, without any anxiety or concern about harming oneself or others.

Students’ needs are also configured based on present conditions, which take into account the changes that have occurred. In this sense, new necessities have emerged that have brought to light a greater awareness of issues and have been perceived in terms of opportunities for improving daily life: having more space for oneself, being independent, being listened to and understood, accepting one’s body, not being considered as numbers, but as people who, as such, live in relationships, experience emotions, and may encounter difficulties. In this regard, one student stated:

“Like many students, I often feel too anchored to the rigidity given by evaluations and teachers’ judgments. I often find myself feeling like just a number, and it seems that teachers are even more interested in our school situation than we are. In my opinion, the will to learn must come from within (with the help of teachers) and should not be forced because this leads to conceiving school as a place where one’s self is oppressed to memorize notions transcribed in books. On the contrary, I would like school to be a place where one can spontaneously engage, and not, as I often see, only to get a positive grade or to obtain approval from the teacher figure.” (S19).

#### The need to develop one’s skills

Secondly, it appeared interesting to bring attention to the positioning through which students have configured current needs through prescriptions for themselves and others. Actions were indicated that, according to the student, must or must not be implemented by those who are required to welcome and respond to these needs.

As previously noted, the need not to be judged, to receive sincere observations about the state of things, to interact with involved and empathetic people, to receive listening, understanding, and comfort is highlighted.

“I would like someone to talk to without seeing me as a strange person, someone you can talk to without being judged. I would like someone who tells me things as they are, without having a certain ‘pity’ for me. At the same time, however, I do not want them to be cold; I want them to truly understand what I’m going through, not because they have to, but because they want to.” (S44).

Moreover, students identify needs through the necessity to implement and internalize some fundamental abilities that can allow them to integrate, be enterprising, know how to make decisions, interact positively with adults, develop a critical point of view, recognize their rights and duties, thus facing the identified instances firsthand.

“We have the need to integrate, to know how to be enterprising, to know how to make complicated decisions, to interact positively with adults since school is the first place where this confrontation occurs. This is why we need to be listened to, to be led towards a path that is not necessarily that of work, but towards the ability to know how to elaborate personal criticism and think with our own heads” (Interv.33).

## Discussion

Among the interpretative repertoires that emerged from students’ descriptions of the professional figure in question, we first find that of “necessity.” As a common background to the narratives, a scenario was traced in which the school psychologist is primarily described as a fundamental professional figure, indispensable to students in the school context. These narrative modes highlight that the presented reality is taken for granted by students, and therefore does not require further verification beyond its mere presence. In this historical period, it simply exists as self-evident and indisputable factuality (Iudici et al., 2021). The perception of the school psychologist as a ‘necessary’ figure reflects a global trend towards greater attention to mental health in schools. This alignment with international practices is highlighted in studies such as that of Kutcher et al. (2016), which examined the integration of mental health in school systems across various countries.

Students have thus proposed a reality in which the psychologist’s intervention proves useful not only in particular situations where moments of impasse and suffering manifest, but especially in the moments of daily life that take place at school, which inevitably bring with them certain challenges. This data confirms the idea of expanding the role of the school psychologist in the school context (Albritton et al., 2019).

This thought assumes particular importance as an expression of an evolution in the conception of the school psychologist whose role, as previously stated, has often been limited to emergency situations (Sheridan and Gutkin, 2000). The emphasis on the role of the school psychologist in students’ daily lives, not just in crisis situations, reflects a paradigm shift observed at an international level. This preventive approach and promotion of well-being aligns with the World Health Organization’s recommendations for mental health in schools (WHO, 2021).

It is therefore fundamental to associate the figure of the psychologist at school not only with the management of difficulties, but also with the promotion of well-being and support for young people who, by nature, face a complex growth period.

Their involvement and full participation in the school system are therefore demanded, so that, thanks to their specific skills, they can closely know and observe the dynamics that unfold among the actors involved, to then propose tailored interventions that take into account, above all, the particular modes of interaction between participants.

Another significant metaphor that emerged is that of the “school psychologist as a source of help and support.” This configuration reveals an awareness of students’ need to be accompanied in their growth path by a figure who can guide and orient them, not only in their educational journey but also in managing the uncertainties and insecurities that arise in everyone’s life (Merrell et al., 2011).

The idea of the ‘school psychologist as a source of help and support’ reflects a global trend towards a more holistic approach to education, integrating emotional and social well-being with academic learning. This alignment is evident in the practices of many countries, such as those in Scandinavia, but also in some Asian countries, known for their innovative approaches to education (Sahlberg, 2015).

With the image of the “problem solver,” students attribute to the psychologist great responsibility and competence in bringing to light awareness regarding self-knowledge and knowledge of others. We can therefore affirm that it is essential to include in the professional’s mandate the sharing of responsibilities in the co-construction of growth paths, so that students themselves can become the architects of the world they inhabit and can, therefore, assume an active position that restores their autonomy and competence.

Through their narratives, students convey a message of suffering with respect to the typical dynamics of the society in which they are immersed, expressing the desire to reclaim their own value, interests, and vulnerabilities, and claiming the legitimization and constancy of the psychologist’s intervention in the school context.

This attitude is also shown in the contrast of stereotypes and prejudices that place limits on interaction with the school psychologist and in the need, therefore, for a change of perspective that can dismantle preconceived ideas of “healthy” or “sick,” which do not allow for experiencing the interaction with the professional figure in a serene, authentic, and genuine way. This data is also found in other research such as Corrigan (2016) and Smith & Applegate (2018). Addressing and dismantling stereotypes and biases associated with school psychologists is a widespread challenge across various cultural settings. Global research, including the work of Bahr et al. (2017), underscores the critical importance of tackling these barriers to enhance the impact of psychological interventions within educational environments.

The narrative repertoires also address stereotypes closely connected to the school psychologist as an adult figure, reporting the problematization of trust towards the latter.

The practiced theory is one in which the adult is associated with the inability to have empathy towards the younger generation and, therefore, the inability to grasp, without judgment, the experience of the young people with whom they interact. This could suggest a distorted mode of communication between young people and adults, laden with misunderstanding, which carries with it some implications, including the closure of the young person towards the adult, influencing the possibility of a request for help. This data confirms the need to better nurture the relationship between adults and students and their communications, as already supported by Sheridan and Garbacz (2022). This data also confirms the idea that such difficulties also arise with respect to a degree of formality present in Italian schools, as already revealed by Cavalli and Argentin (2010). The focus on the relationship between young people and adults and the need to improve communication are themes considered transversal across different cultural contexts. Research conducted in various countries, such as that of Thapa et al. (2013), emphasizes the importance of a positive school climate in fostering conditions for student well-being.

Another narrative repertoire presents the requester as a “strong,” “mature,” “intelligent,” “courageous,” “aware” person, thus through a series of qualities and characteristics that allow them to enter into a relationship with the school psychologist. While on one hand this modality confirms the need for a change of perspective, on the other, it anticipates possible scenarios that could evolve with respect to the modalities of requesting help. For the psychologist, it is therefore fundamental to keep these considerations in mind when designing interventions that take into account, for example, a possible lack of request for help in cases where the student might consider themselves “a person with little or no courage.”

In relation to students’ current needs, a characteristic element is the reference to the past, understood as a safety factor. Students, in fact, recall old habits to emphasize the uncertainty that derives from the continuous changes they must face. In a context where distance and insecurity about the future predominate, on one hand, there is a need to provide skills that students can use to face both their present and any unforeseen events; on the other hand, there is a need for a growth environment not only on the didactic and formative level but also on a personal level, as already reported by the OECD (2020b). The students’ request to be active protagonists in their growth journey and in decisions that concern them reflects a now global need that embodies a movement calling for greater student participation in educational processes. This aligns with certain practices known as ‘student voice’ promoted in many advanced educational systems (Mitra, 2018).

This would allow looking at the present as an opportunity to make space for oneself, one’s abilities and curiosities, and to build positive, satisfying, and fulfilling relationships.

The psychologist can respond to these requests by promoting a change in the structure and organization of the school environment, making it an open, inclusive space that, based on proximity, can grasp the meanings that participants attribute to the dynamics in which they are involved, and ultimately, offer students the possibility to be protagonists in the interactions they experience, and in the interventions and decisions that concern them.

The emphasis on the need to prepare students to face uncertainty and continuous social changes reflects a global concern in contemporary education. Various organizations have highlighted the importance of developing resilience and adaptability skills in students to address the challenges of the 21st century (OECD, 2018).

In the final analysis, the reading of positionings based on prescriptions reveals an attitude whereby students demand the figure of the psychologist, indicating and precisely establishing what the institution must do and how it must move to respond to this need. In this way, students claim and appeal to their right to health which, as defined by the WHO, corresponds to the ability to adapt and self-manage in the face of social, physical, and emotional challenges.

Indeed, a strong propensity and motivation is perceived on the part of students with respect to the intention to face the difficulties and problems identified firsthand.

## Conclusion

Based on the emerged results, it is possible to conclude that the school psychologist is perceived as highly useful and as an indispensable professional figure to support students and other actors in the growth process that unfolds within the school context, bringing awareness to the processual nature of events and actions.

It has proven useful to give voice to students and their needs because only by bringing them to light can they be addressed and managed by the school system.

The research therefore directs its utility to professionals who intend to place their interest and/or carry out their work in the school context, providing important information on how to build an adequate and effective psychological service at school. Through the analysis of narratives and positionings, specifically, the school psychologist can propose intervention modalities that take into account the complexities that unfold through experiences, meanings, thoughts, needs, beliefs, and all the elements that constitute the dense network of interactions in which actors are involved. The work must be characterized by attention and respect for subjectivity through interventions that invite teachers, parents, and students to get in touch with their emotional and cognitive world in a game of mutual recognition that becomes knowledge of self and others.

The professional must therefore move along a continuum that ranges from support in specific situations of distress to promoting the well-being of the actors involved, positioning users’ needs in an expanded epistemological framework, and working on social representations related to the psychologist’s action and on expectations that come from students, parents, and school staff (Castelli, 2022).

School psychology must therefore be characterized by flexibility and versatility, and must aim to detect resources and skills, often already existing but not expressed in the school system. The image that derives from this modality is that of the psychologist as a “catalyst of processes,” who acts as a bridge and connects all the systems at play, making his work efficient thanks to the global perspective he assumes (Toso, 2021).

As American psychologist Robert Pianta (2001) has taught for years, the relationship represents the “keystone” of development and growth, both in typical conditions and in those of risk and vulnerability (Castelli, 2022).

Fundamental to fulfilling this task is the collaboration between the numerous participants in the school context, the possibility of exchanging points of view and reading the situations in which they are immersed: only in this way is it possible to propose different but effective activities for problems that are similar in content but not in the ways in which they manifest.

The research contributes by offering interesting elements that it is hoped can be taken into consideration for a clear and exhaustive definition of the role, work, and functions of the school psychologist.

The study also proves useful in making known the preeminent need to legitimize and regulate the figure of the school psychologist, through the development of an adequate regulatory framework of reference.

### Limitations

In conclusion, it appears necessary to mention the limitations of the present research. The first concerns having focused solely on the students’ point of view, thus excluding all other participants who live in the school context such as principals, teachers, operators, and parents, who obviously assume an equally fundamental role in configuring the reality they inhabit.

A limitation of this research concerns its primary focus on the Italian school context. To compensate for this limitation, in the discussion section, we have sought to establish connections with aspects considered transversal across different cultural contexts.

Another limitation that may have occurred concerns the moment in which this research was carried out, namely a few years after COVID-19, when some students might still have been in an active emotional bubble.

For this reason, the research could be carried forward by including aspects overlooked in this study, including the inclusion of all participants and extension to other regions. The specificities related to territories and the relationships that occur, not only among actors internal to the school system but also those that unfold through territorial services, could therefore be explored in depth.

## Data Availability

The datasets presented in this study can be found in online repositories. The names of the repository/repositories and accession number(s) can be found in the article/supplementary material.
